# Long-term exposure to Myozyme results in a decrease of anti-drug antibodies in late-onset Pompe disease patients

**DOI:** 10.1038/srep36182

**Published:** 2016-11-04

**Authors:** Elisa Masat, Pascal Laforêt, Marie De Antonio, Guillaume Corre, Barbara Perniconi, Nadjib Taouagh, Kuberaka Mariampillai, Damien Amelin, Wladimir Mauhin, Jean-Yves Hogrel, Catherine Caillaud, Giuseppe Ronzitti, Francesco Puzzo, Klaudia Kuranda, Pasqualina Colella, Roberto Mallone, Olivier Benveniste, Federico Mingozzi, French Pompe Registry Study Group, G. Bassez, A. L. Bedat-Millet, A. Behin, B. Eymard, S. Leonard-Louis, T. Stojkovic, A. Canal, V. Decostre, F. Bouhour, F. Boyer, Y. Castaing, F. Chapon, P. Cintas, I. Durieu, A. Echaniz-Laguna, L. Feasson, A. Furby, D. Hamroun, X. Ferrer, G. Solé, R. Froissart, M. Piraud, D. Germain, K. Benistan, N. Guffon-Fouilhoux, H. Journel, P. Labauge, A. Lacour, A. Levy, A. Magot, Y. Péréon, M. -C. Minot-Myhié, A. Nadaj-Pakleza, C. Nathier, D. Orlikowski, N. Pellegrini, P. Petiot, J. Praline, F. Lofaso, H. Prigent, A. Dutry, D. Renard, S. Sacconi, C. Desnuelle, E. Salort-Campana, J. Pouget, V. Tiffreau, D. Vincent, F. Zagnoli

**Affiliations:** 1 University Pierre and Marie Curie, INSERM, UMR974, Paris, France; 2 Paris-Est neuromuscular center, Institute of Myology, Pitié-Salpêtrière Hospital, AP-HP, Paris, France; 3 University Paris Descartes, INSERM, UMR1138, Paris, France; 4 Genethon, INSERM, UMR951, Evry, France; 5 Department of Internal Medicine and Clinical Immunology, DHUI2B, Pitié-Salpêtrière Hospital, AP-HP, Paris, France; 6 Neuromuscular Physiology and Evaluation Lab, Institute of Myology, Paris, France; 7 Department of Metabolic Biochemistry, Necker Hospital, Paris, France; 8 Institute Cochin, INSERM U1016, CNRS UMR8104, Paris, France; 9 University Paris Descartes, Faculty of Medicine, Paris, France; 10 Department of diabetology, Cochin Hospital, AP-HP, Paris, France; 11 Centre de référence de pathologie musculaire Paris-Ouest, Hôpital Henri-Mondor, Créteil; 12 Centre de compétence de pathologie neuromusculaire, CHU Charles Nicolle, Rouen; 13 Centre de référence de pathologie neuromusculaire Paris-Est, institut de Myologie, groupe hospitalier Pitie -Salpetrière, Paris; 14 Service ENMG et Pathologies Neuromusculaires, Hopital Neurologique Pierre Wertheimer, GHE Lyon, Bron; 15 Service de médecine physique et de réadaptation, CHU de Reims; 16 Service de réanimation, CHU de Bordeaux, Bordeaux; 17 Centre de compétence des maladies neuromusculaires, CHU de Caen; 18 Centre SLA et maladies neuromusculaires, CHU de Toulouse-Rangueil, Toulouse, France; 19 Service de médecine interne, centre hospitalier Lyon Sud, Pierre-Bénit; 20 Département de neurologie, hôpitaux universitaires de Strasbourg, hôpital de Hautepierre, Strasbourg; 21 Centre de référence des maladies neuromusculaires rares Rhône-Alpes, Hôpital Nord, CHU de Saint-Etienne; 22 Centre Hospitalo-Universitaire de Montpellier. Hôpital Arnaud de Villeneuve, 34000. Montpellier; 23 Centre de référence des maladies neuromusculaires, CHU de Bordeaux-GH Sud - hôpital Haut-Levesque, Pessac; 24 Centre de biologie et pathologie Est, hospices civils de Lyon, Bron; 25 Service de génétique médicale, Hôpital Raymond-Poincaré, Garches; 26 Unité des maladies métaboliques, département de pédiatrie, CHU de Lyon-GH Est hôpital Femme-Mère-Enfant, Bron; 27 Génétique médicale, centre hospitalier Bretagne-Atlantique, Vannes; 28 Département de Neurologie, CHU de Montpellier, Montpellier; 29 Service de Neurologie D et centre de réference des maladies rares neuromusculaires, C.H.R.U Lille, Hopital Roger Salengro, Lille; 30 Service de pneumologie, centre hospitalier Jacques-Cœur, Bourges; 31 Centre de référence des maladies neuromusculaires Nantes-Angers, Hôtel Dieu, Nantes; 32 Service neurologie, CHU de Rennes, Rennes; 33 Centre de référence des maladies neuromusculaires Nantes/Angers, Service de neurologie, CHU d’Angers, Angers; 34 Service de réanimation et unité de ventilation à domicile, CICIT 805, Hôpital Raymond Poincaré, Garches; 35 Service de soins de suite et de réadaptation neurologie, GHI du Vexin, Aincourt; 36 Centre de référence maladies neuromusculaires de la région Rhône-Alpes, hôpital de la Croix-Rousse, Lyon; 37 Centre de compétences maladies neuromusculaires, CHRU de Tours, Tours; 38 Département d’explorations fonctionnelles, Hôpital Raymond-Poincaré, Garches; 39 CHU de Nîmes, hôpital Caremeau, Nîmes; 40 Centre de réference des maladies Neuromusculaires et SLA, Hôpital Archet1 BP 3079, Nice; 41 Centre de référence des maladies neuromusculaires et de la SLA, Hôpital de la Timone, Aix-Marseille Université, Marseille; 42 Centre de référence des maladies neuromusculaires, CHRU de Lille, Lille; 43 Service de neurologie, groupe hospitalier La Rochelle – Ré – Aunis, La Rochelle; 44 Hôpital d’Instruction des Armées Clermont Tonnerre, Brest, France

## Abstract

Immunogenicity of recombinant human acid-alpha glucosidase (rhGAA) in enzyme replacement therapy (ERT) is a safety and efficacy concern in the management of late-onset Pompe disease (LOPD). However, long-term effects of ERT on humoral and cellular responses to rhGAA are still poorly understood. To better understand the impact of immunogenicity of rhGAA on the efficacy of ERT, clinical data and blood samples from LOPD patients undergoing ERT for >4 years (n = 28) or untreated (n = 10) were collected and analyzed. In treated LOPD patients, anti-rhGAA antibodies peaked within the first 1000 days of ERT, while long-term exposure to rhGAA resulted in clearance of antibodies with residual production of non-neutralizing IgG. Analysis of  T cell responses to rhGAA showed detectable T cell reactivity only after *in vitro* restimulation. Upregulation of several cytokines and chemokines was detectable in both treated and untreated LOPD subjects, while IL2 secretion was detectable only in subjects who received ERT. These results indicate that long-term ERT in LOPD patients results in a decrease in antibody titers and residual production of non-inhibitory IgGs. Immune responses to GAA following long-term ERT do not seem to affect efficacy of ERT and are consistent with an immunomodulatory effect possibly mediated by regulatory T cells.

Pompe Disease (PD) is an autosomal recessive myopathy caused by a deficiency in the lysosomal enzyme acid α-glucosidase (GAA), which results in an abnormal storage of glycogen in several tissues^1^,^2^. The infantile form of PD is the most severe and, if not treated, is associated with early lethality^3^,^4^. The adult form of the disease, known as late onset Pompe disease (LOPD), is compatible with life, although it is associated with progressive deterioration of skeletal muscle function, leading in some cases to significant disability and need for assisted ventilation^5^,^6^. The approval of recombinant human GAA (rhGAA) (Myozyme) for the treatment of Pompe disease resulted in a significant improvement of both life expectancy and quality of life of infantile PD patients^7^, although long-term follow up of children treated with enzyme replacement therapy (ERT) revealed occurrence of symptoms resulting from the incomplete correction of the enzyme deficiency in certain tissues^8^. Approval of ERT for LOPD patients followed that of pediatric subjects few years later, and long-term benefit of ERT in this population is still being assessed^9^,^10^,^11^.

One important common side effect of ERT for Pompe disease is the induction of antibody responses against the infused protein, a phenomenon particularly frequent in infantile patients who are cross-reactive immunological material (CRIM)-negative^4^,^12^, that is associated with lack of efficacy and poor prognosis^13^,^14^. Similarly, despite being CRIM positive, anti-rhGAA antibodies in response to ERT are found also in LOPD patients, although their role is unclear when it comes to the clinical response to enzyme supplementation^15^. In addition to neutralizing antibody responses, life-threatening allergic reactions associated with the production of immunoglobulin (Ig) E specific to the enzyme have been reported to occur following the infusion of rhGAA^16^.

Unlike for infantile PD and for other lysosomal storage disorders^17^,^18^, little is known on the impact of immune responses to rhGAA in LOPD subjects undergoing ERT. Moreover, mechanistic insights into the immunogenicity of rhGAA are lacking. Limited studies in human peripheral blood mononuclear cells (PBMC) showed dose-dependent increase in interferon gamma (IFNγ) and tumor necrosis factor alpha (TNFα) production in CD4^+^ and CD8^+^ T cells in LOPD patients receiving ERT^19^. Additional studies in GAA-*null* mice treated with rhGAA showed high frequency of T cells producing interleukin (IL) 4 in response to rhGAA, highlighting a predominantly T helper (Th) 2-driven immune response^20^,^21^,^22^.

Here we characterized the immune responses to rhGAA in a large cohort of LOPD subjects either receiving long-term ERT or untreated. In this study we demonstrate that rhGAA infusion results in the early production of high-titer antibodies in a subset of subjects, however antibodies appear to drop over time with continuation of ERT. IgG subclass characterization shows production of non-inhibitory antibodies with no evident effect on enzyme activity or uptake, while rhGAA-specific T cell activation profile is consistent with immune modulation, possibly mediated by regulatory T cells.

## Results

### Long-term ERT results in clearance of anti-rhGAA antibodies in LOPD patients

**Figure 1:**
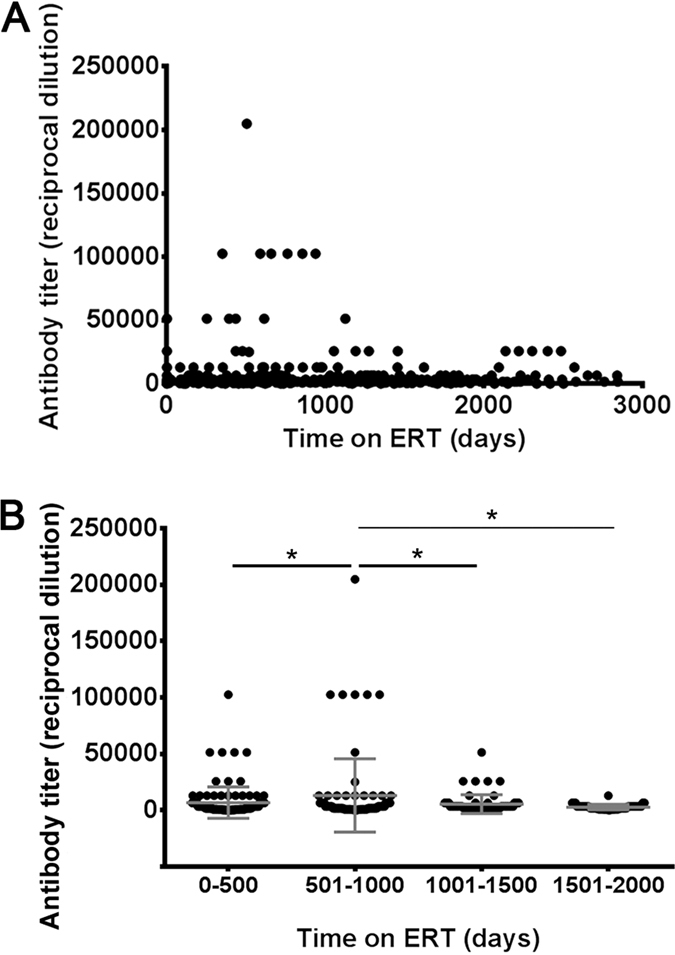
Long-term ERT in LOPD patients results in a decrease of anti-rhGAA antibodies. (**A**) Measurement of anti-rhGAA antibody titers over time (n = 24 subjects). (**B**) Comparison of antibody titers measured in the first 500 days of ERT (n = 108 measurements) with titers measured between 500 and 1,000 days of ERT (n = 82 measurements), 1,000 and 1,500 days of ERT (n = 64 measurements), and 1,500 and 2,000 days of ERT (n = 46 measurements). For each time period, the same number of measurements for each subject was included in the analysis. ANOVA analysis was used to compare the measurements over time (*P < 0.05). Antibody titers are expressed as reciprocal dilution as previously described^10^.

To understand the effect of long-term ERT on humoral responses to rhGAA, anti-rhGAA antibody data from LOPD subjects (n = 24) who received ERT with Myozyme at least 3 years (three antibody measurements per year) and developed a response to the enzyme were collected and analyzed. Of these subjects, five had peak antibody titers ≥1:25,000, twelve had peak antibody titers ≥1:6,400 and <1:25,000, and seven developed peak antibody titers <1:6,400. No clear correlation between GAA gene mutations (Supplementary Table S1) and peak antibody titers was found (data not shown). 17 out of 24 patients showed the highest antibody titer in the first 1,000 days on ERT (Fig. 1A), with one subject reaching a titer of >1:200,000, followed by a decrease to levels close to baseline for most subjects. Statistical analysis of data, performed by comparison of titers measured during the first 500 days of ERT (108 measurements) with those collected between 501 and 1,000 days (82 measurements), between 1,001 and 1,500 days (64 measurements), and between 1,500 and 2,000 days of ERT (46 measurements), confirmed that the changes in antibody titers were significant (Fig. 1B). These results indicate that long-term ERT in LOPD subjects is associated with a decrease of anti-rhGAA antibodies.

### Long-term ERT in LOPD subjects results in development of non-neutralizing anti-rhGAA IgG1 and IgG4

**Figure 2:**
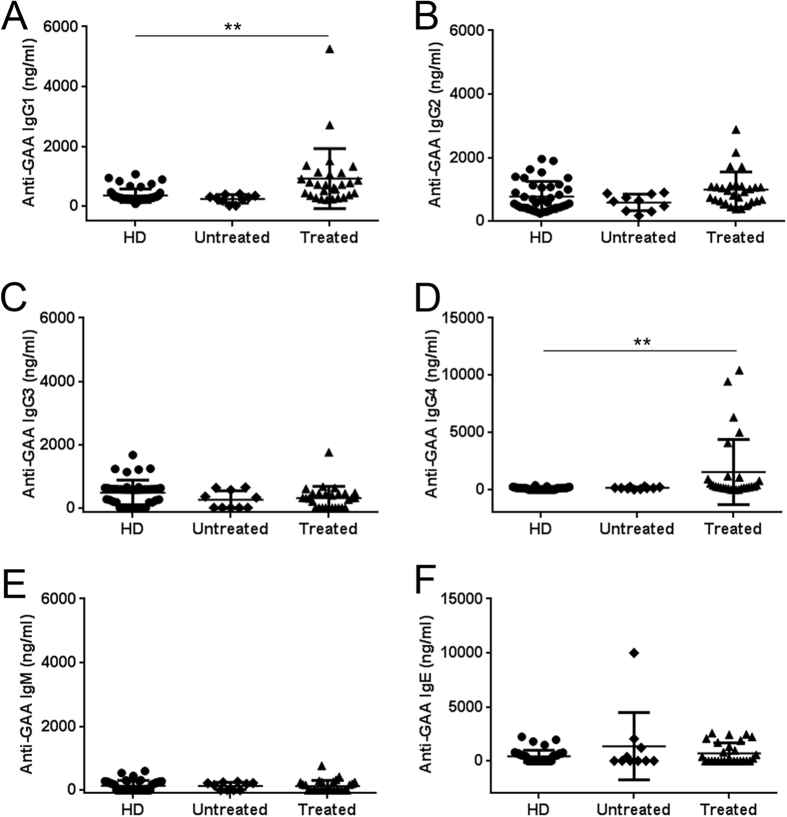
Anti-rhGAA IgG1 and IgG4 are the most prevalent subclasses of antibodies developed in response to long-term ERT. (**A**–**F**) Anti-rhGAA antibodies measured with a capture assay specific for IgG1, IgG2, IgG3, IgG4, IgM, and IgE antibodies. Estimated antibody concentrations are reported for each subject. Average and standard deviation are indicated for each of the study cohorts: LOPD subjects receiving ERT (Treated, n = 28), untreated LOPD subjects (Untreated, n = 10), and healthy donors (HD, n = 43). Unpaired two-tailed t-test was used to compare results across the study cohorts (**P < 0.01).

To better understand the nature of anti-rhGAA antibody responses observed in the LOPD patients enrolled in the study, serum samples were tested using an antibody binding assay specific for anti-rhGAA IgG subclasses, IgM, and IgE (Fig. 2). Healthy donors (HD) and untreated LOPD subjects did not display significant levels of antibodies specific to rhGAA, whereas a subset of LOPD subjects who received long-term ERT had elevated levels of anti-rhGAA IgG1 and IgG4 antibodies (Fig. 2D). No significant levels of IgG2, IgG3, IgM, and IgE antibodies were detected. As IgG4 have been reported in association with inhibitor activity^23^, seven samples, displaying the highest anti-rhGAA antibody titers, were then tested for neutralizing activity on both rhGAA enzyme activity and cell uptake assays and no inhibitory activity was measured (Supplementary Fig. S1A). In agreement with these results, follow up of six-minute walk test (6MWT) and forced vital capacity (FVC) in IgG4 positive subjects showed a similar trend to the subjects from the same cohort (Supplementary Fig. S1B,C, and *vide infra*). These results confirm the presence of anti-rhGAA antibodies in CRIM-positive LOPD subjects even after long-term ERT. They also indicate that repeated exposures to the rhGAA antigen results in production of non-neutralizing antibodies including IgG4, an antibody subclass previously associated with activation of regulatory B cells^24^,^25^.

### DC-mediated restimulation of PBMCs results in detection of T cell reactivity to rhGAA

**Figure 3:**
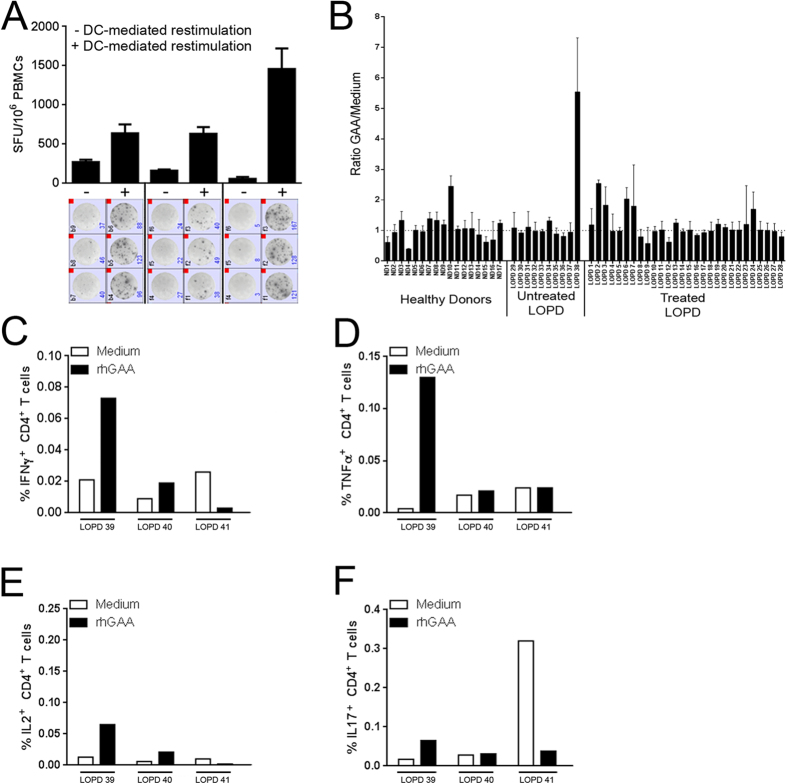
T cell reactivity to rhGAA is detectable after restimulation of PBMCs with the antigen. (**A**) Comparison of IFNγ ELISpot count in PBMCs isolated from LOPD subjects receiving ERT before and after DC-mediated restimulation. Cells were either directly plated in the ELISpot assay (−) or restimulated *in vitro* with 10 μg/ml of rhGAA prior to the assay (+). Results are expressed in spot forming units (SFU) per 10^6^ cells. Results are reported as average of triplicate testing +/− standard deviation. Images of the test wells in the IFNγ ELISpot assay are shown below the histogram plot. (**B**) Combined results for all subjects screened with the IFNγ ELISpot assay after PBMC restimulation. PBMCs from LOPD subjects receiving ERT (Treated, n = 28), untreated LOPD subjects (Untreated, n = 10), and HD (n = 15) were tested in the assay. Results are expressed as ratio between SFU/10^6^ cells in rhGAA-restimulated cells vs. medium control. (**C**–**F**) PBMCs from LOPD subjects receiving ERT (n = 3) were restimulated with 10 μg/ml of rhGAA for 48h and then stained intracellularly for IFNγ, TNFα, IL2, and IL17. Results are reported as percentage of CD4^+^ T cells secreting a given cytokine.

IgG4 production has been associated with induction of immunological tolerance^24^. To test whether a similar mechanism mediated the observed decrease in anti-GAA antibody titers in LOPD subjects undergoing ERT, and to study T cell reactivity to rhGAA in treated and untreated LOPD subjects, an IFNγ ELISpot assay specific for the enzyme was established. Initial screening of PBMCs isolated from treated LOPD subjects revealed lack of reactivity to rhGAA, even in subjects with detectable anti-rhGAA antibodies (data not shown). To enhance T cell reactivity to rhGAA, we used a dendritic cell (DC) activation protocol optimized to study low-frequency T cells^26^. Following restimulation with rhGAA and specific cytokines and chemokines prior to the ELISpot assay, we were able to measure production of IFN-γ in response to rhGAA in some subjects (Fig. 3A). Five out of twenty-eight treated LOPD subjects showed detectable T cell reactivity to rhGAA, however no correlation was found between production of IFNγ and anti-rhGAA antibody titers (Fig. 3B). A positive T cell response to rhGAA was detected also in one untreated LOPD subject and one HD, an observation previously made for other antigens such as coagulation factor VIII in HD and in hemophilia A subjects^27^. Additional analyses of treated LOPD PBMCs restimulated *in vitro* with rhGAA for 48 hours followed by intracellular cytokine staining (ICS), showed a complex pattern of activation of CD4^+^ T cells, with production of IFNγ, TNFα, IL2, and IL17 in some subjects (Fig. 3C–F). No response to rhGAA was detectable in CD8^+^ T cells (data not shown). These results indicate that circulating GAA-reactive T cells can be found in peripheral blood, although detectable only after restimulation with the antigen^28^.

### IL2 is the cytokine signature of T cell reactivity to rhGAA in treated LOPD subjects

**Figure 4:**
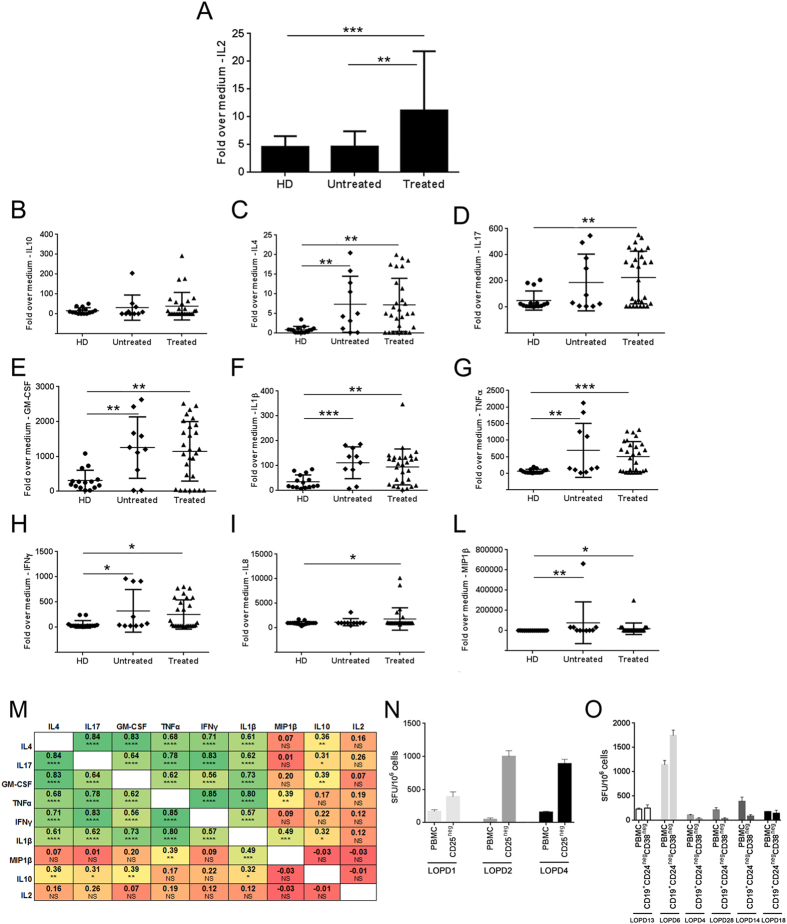
Cytokine profiling of supernatant from PBMCs restimulated with rhGAA. Supernatants from cells restimulated with rhGAA were collected after 48 hours of restimulation *in vitro* and assayed for cytokine and chemokine production. (**A**) Levels of IL2 measured in conditioned media in LOPD subjects receiving ERT (Treated, n = 28), untreated LOPD subjects (Untreated, n = 10), and healthy donors (HD, n = 17). Error bars represent the standard deviation of the mean. Mann-Whitney test was used to compare data across the study groups. (**B**–**L**) Cytokine and chemokine concentration in media measured with the Luminex array technology; shown are individual values measured in LOPD subjects receiving ERT (Treated, n = 28), untreated LOPD subjects (Untreated, n = 10), and healthy donors (HD, n = 17). Error bars represent the average of a cohort +/− standard deviation. Mann-Whitney test was used to compare data across the study groups. (**M**) Pearson correlation matrix comparing measurements of cytokine and chemokine production in responses to rhGAA in treated (n = 28) and untreated (n = 10) LOPD subjects, and HD (n = 15). Numbers in the table represent the correlation coefficient between two variables with the relative p value (t-test). (**N**) Depletion of CD25^+^ in PBMCs from treated LOPD subjects. The untouched CD25^neg^ PBMC fraction was co-cultured with autologous DCs pulsed with rhGAA antigen or unpulsed DCs as negative control, followed by IFNγ ELISpot. Results of the IFNγ ELISpot assay are shown as average of spot forming units (SFU) per 10^6^ cells plated in the assay +/− standard deviation of triplicate testing. (**O**) Sorted untouched CD19^+^CD24^neg^CD38^neg^ PBMC fraction from treated LOPD subjects co-cultured with autologous DCs pulsed with rhGAA antigen (or negative control) followed by IFNγ ELISpot. Results of are shown as average SFU/10^6^ cells +/− standard deviation of triplicate testing (*P < 0.05; **P < 0.01; ***P < 0.001; ****P < 0.0001, NS, not significant).

To better define the profile of T cells reactive to rhGAA in all subjects studied, we collected supernatant from PBMCs after DC-mediated restimulation and tested them against an array of cytokines and chemokines using the Luminex platform (Fig. 4 and Supplementary Fig. S2). In this assay, significantly elevated levels of IL2 were detected only in treated LOPD subjects (Fig. 4A). IL10 was also slightly upregulated in subjects receiving ERT, although not at significant levels (Fig. 4B). Conversely, significant amounts of cytokines were found in both treated and untreated LOPD subjects compared with HD (Fig. 4C–L). A Pearson correlation matrix was used to analyze the data deriving from the cytokine and chemokine production in treated and untreated LOPD subject and the data deriving from the HD, confirming the presence of a correlation among cytokines in responses to rhGAA in both treated and untreated LOPD subjects (Fig. 4M). These results may reflect the proinflammatory state associated with the disease^19^,^29^, which was previously described in infantile Pompe patients^30^. Alternatively, the reactivity detected against rhGAA in both treated and untreated LOPD patients may reflect an underlying immune response resulting from the expression of the endogenous mutant GAA protein.

The secretion of IL2 after DC-mediated restimulation only in treated LOPD subjects possibly reflects a state of anergy of reactive T cells deriving from long-term exposure to rhGAA^31^. Alternatively, the detection of slightly elevated levels of IL10 in treated LOPD subjects may also be evidence of equilibrium between proinflammatory and tolerogenic signals. To address this point, the untouched CD25^neg^ fraction of PBMCs was isolated from three LOPD subjects displaying high anti-rhGAA IgG4 levels. Following a 48-hour restimulation with rhGAA, both the untouched PBMC and the CD25^neg^ fraction of PBMC was co-cultured with autologous DCs pulsed with rhGAA. Cells were tested for reactivity to rhGAA in an IFNγ ELISpot assay, showing enhanced T cell reactivity to rhGAA in the CD25^neg^ fraction of PBMC (Fig. 4N), suggesting that the induction of peripheral tolerance contributed to the unresponsiveness to the rhGAA antigen. Similarly, to investigate the role of suppressive IL10-expressing B cells^24^,^32^, we depleted CD19^+^CD24^hi^CD38^hi^ B cells in PBMC collected from six treated LOPD subjects. The remaining untouched PBMC fraction was co-cultured with autologous DCs pulsed with rhGAA antigen and then tested for reactivity to rhGAA in an IFNγ ELISpot assay. Depletion of regulatory B cells resulted in a slight enhancement of T cell reactivity to rhGAA in only 1/6 subjects (Fig. 4O), not supporting the involvement of CD19^+^CD24^hi^CD38^hi^ B cells in the observed hyporesponsiveness to rhGAA.

### Infusion of rhGAA in LOPD subjects is associated with systemic upregulation of cytokines and chemokines

**Figure 5:**
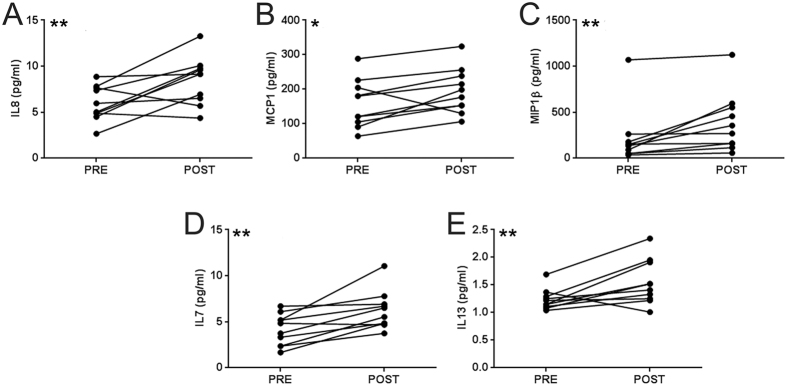
Serum cytokine and chemokine profiling of LOPD subjects before and after ERT. (**A**–**E**) Luminex cytokine and chemokine measurements before (PRE) and immediately after (POST) rhGAA administration (over the course of four hours) in LOPD subjects (n = 10). Shown are cytokines for which a significant change in serum levels was measured at the end of ERT. Paired t-test was used to compare data analysis PRE vs. POST infusion (*P < 0.05; **P < 0.01).

To test whether rhGAA infusion is associated with systemic activation of the immune system, we performed a study in ten LOPD subjects undergoing ERT in which we monitored serum cytokines and chemokine levels. For each subject, a serum sample was collected immediately before the infusion of rhGAA and one additional sample was collected at the end of the infusion, after flushing the intravenous line with saline solution. rhGAA administration resulted in a significant upregulation of several cytokines and chemokines, including IL8, monocyte chemotactic protein 1 (MCP1), macrophage inflammatory protein 1 beta (MIP1β), IL7, and IL13 (Fig. 5). Additional cytokines were upregulated, although the changes measured were not significant; these included IL6, TNFα, IL1β, IFNγ, IL4, IL5, and granulocyte macrophage colony-stimulating factor (GM-CSF) (Supplementary Fig. S3). No clear correlation between reactivity to rhGAA and baseline measurements of humoral or cellular responses to rhGAA was evident in the subjects studied (not shown). These results indicate that administration of rhGAA results in early activation of immunity.

### Long-term clinical outcome of ERT does not correlate with immune responses to rhGAA

**Figure 6:**
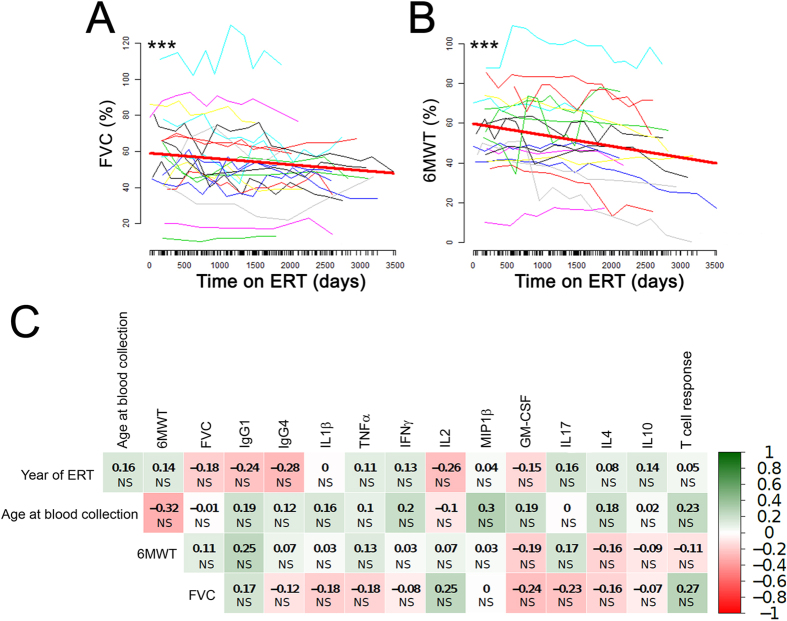
Clinical outcome measures and statistical analysis of results. (**A**) % forced vital capacity (FVC) measurements, expressed as % predicted in healthy individuals, over a period of 4 years in 21 treated LOPD subjects; the red line represents the average evolution of the measurement. The estimated slope is −1.16% per year; the estimated intercept is 58.9%; p value is given by linear mixed models (see Methods). The following equations were used to calculate the % FVC: [FVC predicted for males = 0.060 * height − 0,0214 * age – 4.65]; [FVC predicted for females = 0.0491 * height − 0.0216 * age – 3.59]; [FVC (%) = FVC observed/FVC predicted * 100] (**B**) Six-minute walk test (6MWT), expressed as % predicted in healthy individuals, measured over a period of 4 years in 19 subjects, the red line represents the average evolution of the measurement. The estimated slope is −2.06% per year; the estimated intercept is 59.6%; p value is given by linear mixed models (see Methods) (***P < 0.001). The following equations were used to calculate the % 6MWT: [6MWT predicted for males = −309–5.02 * age − 1.76 * weight + 7.57 * height]; [6MWT predicted for females = 667–5.78 * age − 2.29 * weight + 2.11 * height]; [6MWT (%) = 6MWT observed/6MWT predicted * 100]. (**C**) Spearman correlation of measurements of immune responses to rhGAA with clinical outcome measures recorded at the time of blood collection. The numbers in the table represent the correlation coefficient between two variables with the relative p value (t-test) (NS, not significant).

Follow-up measurements of six-minute walk test (6MWT) and forced vital capacity (FVC) in the cohort of treated LOPD subjects (Fig. 6A,B respectively) showed an initial improvement or stabilization of both measurements followed by a downward trend. This finding, which possibly reflects a worsening of the disease despite ERT in a subset of patients, will need to be confirmed in larger studies. Notably, a recently published meta-analysis of clinical trials involving LOPD patients undergoing ERT also showed a marked improvement in 6MWT and FVC in the first year of treatment, followed by a subsequent stabilization or decline in performance^11^.

In our analysis, no correlation was found between the evolution of FVC and 6MWT and the measurements of immune responses to rhGAA after long-term ERT (Fig. 6C), consistent with the fact that no neutralizing antibody response was detected after long-term ERT. These results confirm that, after long-term ERT, no relationship between clinical endpoints of disease progression and immune responses to rhGAA is found.

## Discussion

Immunogenicity of protein-replacement therapeutics represents a major hurdle in the treatment of genetic^33^ and acquired^34^ disorders. Several factors contribute to shape the immune responses to an antigen, including the genetic background of the host^4^,^35^, the immunogenicity of the therapeutic protein^36^, polymorphisms in genes regulating immune responses^37^, and presence of contaminants in the drug substance that can exert an adjuvant effect on immune responses^36^. Additionally, underlying inflammation associated with some diseases can enhance immune responses against protein and gene therapeutics^38^,^39^. To date, the mechanisms driving the immunogenicity of rhGAA in ERT for Pompe disease are largely unknown.

Here we presented a comprehensive characterization of immune responses to rhGAA in a cohort of subjects affected by LOPD, either untreated or undergoing long-term ERT. The data presented demonstrate that in this patient population, despite the presence of residual GAA antigen endogenously expressed^40^, antibodies responses are elicited by ERT, in some cases reaching high titers (Fig. 1A and refs 15 and 41). Unlike for other genetic diseases^35^, no clear correlation with the underlying mutation in the disease-causative gene was found, reflecting the fact that LOPD subjects are typically CRIM-positive, and suggesting that other genetic factors^42^ may contribute to shape the response to rhGAA. To this end, secretion of cytokines in response to rhGAA by PBMC collected both from treated and untreated LOPD subjects indicate the existence of an underlying proinflammatory condition associated with the disease, regardless of ERT. This is possibly the consequence of impaired autophagy^43^ that characterizes Pompe disease and other lysosomal storage diseases^44^,^45^. Alternatively, as previously observed in Duchenne muscular dystrophy patients^38^, residual expression of the endogenous GAA in these subjects may also result in an increased immunogenicity of rhGAA. These results were also confirmed *in vivo*, as we showed that infusion of rhGAA in LOPD patients triggered immediate systemic secretion of several cytokines and chemokines.

In our cohort of subjects, despite the fact that some subjects reached anti-rhGAA antibody titers >1:200,000 during the first ~1,000 days of ERT, long-term exposure to rhGAA in the absence of immunomodulatory interventions resulted in a marked decrease in antibody levels, with no late occurrence of antibodies, except for non-neutralizing IgG1 and IgG4. This outcome of ERT is markedly different that observed in infantile Pompe patients, who need adjuvant immunomodulatory therapy to prevent or eradicate immune responses to rhGAA^13^,^46^,^47^.

Differently from ERT in hemophilia A, where detection of IgG4 is typically associated with presence of inhibitor^23^,^48^,^49^, the presence of increased levels of non-neutralizing IgG4 antibodies, in the absence of IgE antibodies, in treated LOPD subjects compared with HD and untreated LOPD subjects possibly reflects the desensitization to the immunogen^24^,^50^,^51^,^52^. Alternatively, the production of GAA-specific IgG4 may simply reflect the chronic exposure to antigen^53^. Whether the residual anti-rhGAA IgG titers found in our patient cohort affect clearance or bioavailability of the protein, remain to be established^54^.

Immune tolerance associated with ERT has been previously described in humans affected by diseases like mucopolysaccharidosis type I^55^,^56^ and hemophilia B^49^. Studies in mice^20^,^21^ support the idea that CD4^+^ T helper cells are key to the development of humoral responses to rhGAA, confirmed by the fact that targeting these cells with non-depleting monoclonal antibodies results in avoidance of antibody formation to the protein^57^. In our study, detection of CD4^+^ T cell reactivity only after mature DC-mediated restimulation^26^ of PBMCs highlights a state of hyporesponsiveness of T cells, which can produce large amounts of proinflammatory cytokines when encountering the rhGAA antigen in the context of the appropriate stimulus. IL2, in particular appears to be the cytokine signature in LOPD subjects on ERT. Production of IgG4 antibodies has been associated with the presence of IL10-secreting B regulatory 1 (Br1) cells^24^. Detection of slightly elevated levels of IL10 following PBMC restimulation may indicate the existence of some mechanism of active suppression of immune responses to rhGAA that contributes to the development of immune tolerance to the antigen. Indeed, depletion of CD4^+^CD25^+^ T cells from PBMCs did resulted in enhanced T cell reactivity to rhGAA, while little to no effect was observed when CD24^hi^CD38^hi^ B cells^32^ were depleted. While these results are preliminary, they support the role of regulatory T cells in the development of peripheral tolerance to rhGAA following ERT. More detailed studies will also help to fully address the role of CD73^−^CD25^+^CD71^+^ Br1 cells^24^ in Pompe disease patients undergoing ERT.

Finally, it is not surprising that the measurements of immune responses after long-term exposure to ERT do not correlate with the longitudinal analysis of 6MWT and FVC. This is likely to be the results of multiple factors, including (1) all subjects after long-term ERT had low residual levels of anti-rhGAA antibodies which were not neutralizing, (2) the fact that the immune responses measured were more consistent with a state of unresponsiveness, rather than immunity, to rhGAA and (3) the fact that the immune assays were performed far in time from the peak of antibody responses to rhGAA.

In summary, exposure to rhGAA via ERT in LOPD subjects results in early development of antibodies to the protein, which subsequently mostly disappear over time. The presence of residual levels of non-neutralizing rhGAA-specific IgG4 antibodies, and the T cell activation profile consistent with hyporesponsiveness to the antigen, reflect an immunomodulatory effect mediated by the long-term exposure to the antigen, possibly via the induction of regulatory T cells. These data indicate that antibody formation induced by long-term ERT is not a major concern in LOPD patients. Immune tolerance induction protocols based on high-dose antigen exposure^58^,^59^ and/or pharmacological interventions^60^ are valuable tools to manage detrimental humoral immune responses encountered in ERT. Conversely, in LOPD patients, acute reactions to the enzyme are among the most relevant immune complications of ERT^61^,^62^, thus not warranting the use of immunomodulatory strategies to eradicate anti-GAA antibodies.

## Materials and Methods

### Human samples

LOPD subjects included in this study were part of the French registry of adult Pompe disease patients. Approvals from the competent health authorities and the local Ethical Committee of the Pitie-Salpetriere Hospital, Paris, were obtained prior to the initiation of the study (CNIL N/Ref MMS/CWR/AR155497; CCTIRS N: 14.520; CCP approval 25/06/2014). Informed consent was given by each participant prior to inclusion in the study. All the experiments were carried out in accordance with the relevant guidelines for clinical investigation in France and Europe. Study inclusion criteria were diagnosis of PD defined as deficiency of GAA enzyme activity measured in blood or skin fibroblasts. PD diagnosis was confirmed by *GAA* gene sequencing. Exclusion criteria were age older than 80 years, ongoing or recent (less than 3 months) immunosuppressive treatment, malignancy not in remission, immunodeficiency, or autoimmunity. Blood samples were collected via venipuncture in heparin tubes and used to isolate serum and PBMCs.

Medical history was collected at the time of study inclusion, which included measurements of disease progression such as 6MWT and FVC^9^,^10^, for which data were collected through validated testing across all clinical centers participating to the study. Historical measurements of anti-rhGAA antibodies were also collected. Samples from 28 LOPD subjects receiving biweekly infusion with rhGAA at 20 mg/kg (Treated LOPD) were collected. Treated LOPD (average age 58.2 years, standard deviation 2.4 years; median 57.5 years) were on ERT for 6.5 years on average (standard deviation 2.4; median 7), 13 males and 15 females participated to the study. Additional samples from 10 LOPD subjects (4 males and 6 females) who were not on ERT (Untreated LOPD, average age 53.4 years, standard deviation 17.2 years; median 48 years) were also collected, together with 43 healthy donors (HD). Almost all the LOPD subjects carried the mutation c.32-13T > G in at least one allele. Details of the LOPD subjects enrolled in this study are given in Supplementary Table S1. De-identified HD blood samples (n = 43) were collected through the French blood bank (Etablissement Française du Sang, EFS). De-identified PD patient fibroblasts lacking GAA activity were obtained from the Coriell Institute repository (Camden, NJ, USA) and used in the rhGAA uptake assay.

### Antibody assays

Antibody titers over time in treated subjects were determined as previously described^10^. To study the IgG subclasses specific to GAA, 96-well Nunc Polysorp Immunoplate microplates (Dutscher, Paris, France) were coated with rhGAA to a final concentration of 1 μg/ml. A standard curve made of purified human IgG (Gamunex, Grifolds, Meyreuil, France), purified human IgG1 (Life Technologies, Saint Aubin, France), IgG2, IgG3, IgG4, IgE (Abcam, Paris, France), or IgM (Sigma-Aldrich, Lyon, France) was added directly to the plates. Plates were coated overnight at 4 °C. The next day, after blocking the plates, serum samples were added at dilutions of 1:10 and 1:20 in duplicate and incubated overnight at 4 °C. Monoclonal anti-human Ig biotin-conjugated antibodies specific for each subclass (all from Sigma-Aldrich, Lyon, France) were added to the plates. Binding of the secondary antibodies was detected with alkaline phosphatase-conjugated streptavidin (Sigma-Aldrich, Lyon, France). The enzymatic reaction was developed with p-nitrophenyl phosphate (Sigma-Aldrich, Lyon, France) substrate and color development was read at 405 nm using a microplate reader (MRX Relevation, DYNEX Technologies, Denkendorf, Germany). Anti-rhGAA antibody concentration was determined against the Ig subclass-specific standard curve using 4-parameters regression.

To evaluate the neutralizing activity of serum anti-rhGAA antibodies, a GAA activity assay was established as previously described^63^.

### Short-term T cell restimulation with dendritic cell (DC) induction and cell selection

PBMCs were restimulated *in vitro* according to a protocol designed to enhance detection of antigen-specific T cell responses^26^. PBMCs from the three cohorts studied were stimulated with rhGAA to a final concentration of 10 μg/ml, or medium without antigen. Cells recovered after the short-term restimulation protocols were used to perform antigen-specific T cell assays.

CD25^+^ T cell depletion was performed prior to restimulation of PBMC with antigen using magnetic beads conjugated with anti-CD25 antibodies (Miltenyi Biotec, Bergisch Gladbach, Germany). Flow cytometry was used to assess the efficacy of depletion. PBMC depleted of CD19^+^CD24^hi^CD38^hi^ B cells were obtained by staining cells with anti-CD19, CD24, and CD38 antibodies (all BD Biosciences, Le Pont de Claix, France) following by sorting on a FACSAria (BD Biosciences).

### Antigen-specific T cell assays

The IFNγ ELISpot assay was performed as previously described^64^. rhGAA was used at a concentration of 10 μg/ml, medium only served as negative control, and a mix of 0.05 μg/ml of phorbol 12-myristate 13-acetate (PMA, Sigma-Aldrich, Lyon, France) and 1 μg/ml of ionomycin (Sigma-Aldrich, Lyon, France) was used as positive control. All antigens and controls were tested in triplicate.

Intracellular cytokine staining (ICS) was performed by incubating PBMCs from treated LOPD with rhGAA protein (10 μg/ml). Brefeldin A (Sigma-Aldrich, Lyon, France) and monensin (GolgiStop, BD Biosciences, Le Pont de Claix, France) were added to the cultures at a concentration of 1 μg/ml and 2 μM, respectively. A mix of PMA (0.05 μg/ml) and ionomycin (1 μg/ml) was used as positive control in the ICS assay. At a 4-hour incubation with the antigen, cells were harvested and a mix of fluorescently-labeled antibodies specific for the surface markers CD4 (PerCP-Cy5.5, 1:100, eBioscience, Paris, France), and CD8 (Alexa Fluor-700, 1:100, eBioscience, Paris, France), was added to the cells. An amine-reactive aqua dye (Life Technologies, Saint Aubin, France) was used as viability stain. Cells were then fixed and permeabilized with Cytofix/Cytoperm (BD Biosciences Le Pont de Claix, France) and intracellular staining was performed using antibodies against IL2 (PE-Cy7, 1:100 dilution, eBiosciences, Paris, France), IL17 (BV785, 1:20 dilution, BD Biosciences, Paris, France), IFNγ (PE, 1:100 dilution, eBiosciences, Paris, France), TNFα (APC, 1:100 dilution, BD Biosciences, Le Pont de Claix, France). Cells were acquired on a BD LSRFortessa flow cytometer (BD Biosciences, Le Pont de Claix, France). Data analysis was performed using Flowjo software version 10 (Tree star, Ashland, OR, United States).

### Cytokine and chemokine quantitation

Supernatants from PBMC restimulation *in vitro* and serum samples from LOPD subjects were used for the detection of cytokines and chemokines using a multiplexed bead immunoassay based on the Luminex technology. The levels of 17 cytokines (IL1β, IL2, IL4, IL5, IL6, IL7, IL8, IL10, IL12, IL13, IL17, G-CSF, GM-CSF, IFNγ, MCP1, MIP1β, and TNFα) were measured with the Bio-Plex Pro human cytokine 17-plex immunoassay (BioRad, Marnes-la-Coquette, France) following the manufacturer’s instructions. A Bioplex 200 system (BioRad) was used to analyze the samples.

### Statistical analysis

All data are shown as mean value +/− standard deviation. Data analysis was performed as indicated in figure legends using GraphPad Prism version 6 (GraphPad Software Inc., La Jolla, CA, USA). P values lower than 0.05 were considered significant.

Hierarchical clustering of the levels of cytokines and chemokines in HD and PD patients was performed with the StatistiXL software (Digital River, UK) using the Pearson correlation coefficient.

Data deriving from the immune assays (anti-rhGAA IgG levels, cytokine profiles, IFNγ ELISpot assay) and the latest measurements of clinical endpoints were used to calculate the correlation matrix using the Spearman coefficient with the free R software (Version 2.15.2, 32) and the Psych package (Version 1.5.8, Procedures for Personality and Psychological Research, Northwestern University, Evanston, Illinois, USA, http://CRAN.R-roject.org/package=psych). In both correlation matrices the correlation coefficient r was transformed to t using the formula t = r × [√(n − 2)/√(1 − r2)] for statistical testing and the p value adjusted for multiple comparisons using the false discovery rate method.

Only LOPD with a minimum of four years of clinical follow-up for 6MWT and FVC were included in the longitudinal analysis of clinical data. Subjects with a follow-up of <4 years or who started and then discontinued ERT were excluded from the analysis (FVC, n = 21; 6MWT, n = 19). A linear mixed model was used to take into account the within-patient correlation and the between-patient variability as previously described^65^ with the free R software (Version 2.15.2, 32) and the nlme package (Version 3.1-125, Linear and Nonlinear Mixed Effects Models, http://CRAN.R-project.org/package=nlme).

## Additional Information

**How to cite this article**: Masat, E. *et al*. Long-term exposure to Myozyme results in a decrease of anti-drug antibodies in late-onset Pompe disease patients. *Sci. Rep.* **6**, 36182; doi: 10.1038/srep36182 (2016).

**Publisher’s note:** Springer Nature remains neutral with regard to jurisdictional claims in published maps and institutional affiliations.

## Supplementary Material

Supplementary Information
