# Supplementary material for: Long-term exposure to Myozyme results in a decrease of anti-drug antibodies in late-onset Pompe disease patients
Source: Sci Rep. 2016 Nov 4;6:36182. doi: 10.1038/srep36182 (PMC5096052; doi:10.1038/srep36182)
Supplement: Supplementary Information [file srep36182-s1.pdf]

Long-term exposure to Myozyme results in a decrease of anti-drug antibodies in late-onset Pompe disease patients

Elisa Masat[1], Pascal Laforêt[1,2], Marie De Antonio[3], Guillaume Corre[4], Barbara Perniconi[2], Nadjib Taouagh[2], Kuberaka Mariampillai[5], Damien Amelin[1], Wladimir Mauhin[1], Jean-Yves Hogrel[6], Catherine Caillaud[7], Giuseppe Ronzitti[4], Francesco Puzzo[4], Klaudia Kuranda[1], Pasqualina Colella[4], Roberto Mallone[8], Olivier Benveniste[1,5], Federico Mingozzi[1, 4*], French Pompe Registry Study Group[#]

[1]University Pierre and Marie Curie, INSERM, UMR974, Paris, France
[2]Paris-Est neuromuscular center, Institute of Myology, Pitié-Salpêtrière Hospital, AP-HP, Paris, France
[3]University Paris Descartes, INSERM, UMR1138, Paris, France
[4]Genethon, INSERM, UMR951, Evry, France
[5]Department of Internal Medicine and Clinical Immunology, DHUI2B, Pitié-Salpêtrière Hospital, AP-HP, Paris, France
[6]Neuromuscular Physiology and Evaluation Lab, Institute of Myology, Paris, France
[7]Department of Metabolic Biochemistry, Necker Hospital, Paris, France
[8]Institute Cochin, INSERM U1016, CNRS UMR8104, Paris, France; University Paris Descartes, Faculty of Medecine, Paris, France; Department of diabetology, Cochin Hospital, AP-HP, Paris, France
[#]Full list appears at the end of the manuscript

	LOPD	Age	Sex	Years of ERT	Mutations		Clinical Outcome	
					Allele 1	Allele 2	6MWT (meters)	FVC (%)
**Treated**	1	69	M	8	c.-32-13T>G	c.3G>A	467	61
	2	68	F	5	c.-32-13T>G	c.1629C>G	275	116
	3	72	F	4	c.-32-13T>G	c.2481+102_2646+31del	264	56
	4	50	M	9	c.-32-13T>G	c.-32-13T>G	140	34
	5	57	M	2	c.-32-13T>G	c.546+1G>T	461	49
	6	65	M	8	c.-32-13T>G	c.2481+102_2646+31del	95	36
	7	58	F	9	c.-32-13T>G	c.2041-1G>A	N/A*	14
	8	54	M	8	c.-32-13T>G	c.1888+1G>A	300	33
	9	34	F	6	c.1655T>C	c.1688A>T	452	76
	10	80	M	7	c.-32-13T>G	c.1927G>A	220	44
	11	57	M	9	c.-32-13T>G	c.525del	210	31
	12	45	M	5	c.-32-13T>G	c.655G>A	301	13
	13	30	F	7	c.-32-13T>G	c.525del	520	50
	14	69	F	9	c.-32-13T>G	c.118C>T	200	51
	15	54	F	8	c.-32-13T>G	c.1717A>C	229	39
	16	61	F	7	c.-32-13T>G	c.693-1G>C	392	52
	17	49	F	2	c.-32-13T>G	c.1548G>A	314	54
	18	66	M	9	c.-32-13T>G	c.1047del	290	45
	19	57	M	8	c.-32-13T>G	c.1819_1836del	273	77
	20	63	F	9	c.1748 C>T	c.2014C>T	10	57
	21	49	F	8	c.-32-13T>G	c.1819_1836del	N/A*	59
	22	46	F	3	c.-32-13T>G	c.1819_1836del	73	68
	23	77	M	2	c.-32-13T>G	c.925G>A	120	40

	24	48	F	9	c.-32-13T>G	c.2481+102_2646+31del	180	67
	25	67	M	4	c.-32-13C>G	c.-32-3C>G	172	49
	26	65	F	5	c.-32-13T>G	c.1548G>A	N/A*	57
	27	71	M	0.3	c.-32-13T>G	c. 2608C>T	348	53
	28	49	F	6	c.-32-13T>G	c.799-803delinsA	251	109
**Untreated**	29	50	F	N/A	c.-32-13T>G	c.2481+102_2646+31del	354	116
	30	37	M	N/A	N/A	N/A	487	80
	31	45	F	N/A	c.-32-13T>G	c.1636+1G>C	457	99
	32	45	F	N/A	c.-32-13T>G	c.1888+1G>A	533	77
	33	79	F	N/A	c.-32-13T>G	c.1560C>G	312	113
	34	46	M	N/A	c.-32-13T>G	c.1888+1G>A	383	73
	35	26	M	N/A	c.-32-13T>G	c.655G>A	645	101
	36	69	F	N/A	c.-32-13T>G	c.854C>G	252	87
	37	62	F	N/A	c.-32-13T>G	c.2481+102_2646+31del	475	94
	38	75	M	N/A	c.-32-13T>G	c.1560C>G	246	N/A

N/A, not available; *, unable to perform the test

Supplementary Table S1. Characteristics of the study cohorts of treated and untreated LOPD subjects. Patients ID, age of patients, sex, years of ERT, mutations (the nucleotide numbering reflects cDNA numbering with +1 corresponding to the A of the ATG initiation codon in RefSeq NM_000152.3; the amino acid numbering is according to NP 000143.2) and clinical outcome (6MWT and FVC) at the lastest follow up visit.

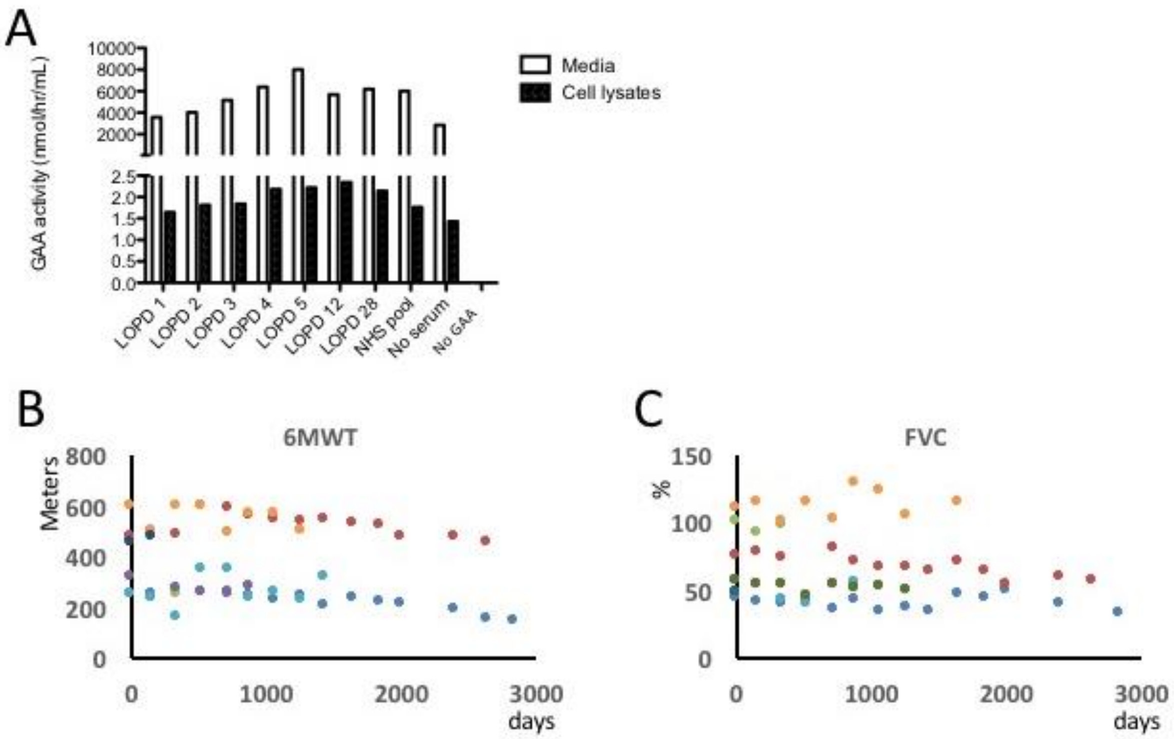

Supplementary Figure S1. Anti-GAA neutralizing antibody assay and follow up of 6MWT and FVC in LOPD subjects with high anti-GAA IgG4 titers. (A) GAA uptake inhibition assay, GAA activity levels are shown in medium and cell lysates following incubation of Pompe fibroblasts with GAA and LOPD sera positive for anti-GAA IgG4 (LOPD1, 2, 3, 4, 5, 12, and 28), pooled healthy donor serum samples (NHS pool), medium only (No serum). Cells incubated with medium without GAA (No GAA) were used as negative/background control. Shown is the results of one of three replicate experiments. (B) 6MWT follow up in LOPD1, 2, 3, 4, 5, 12, and 28. (C) FVC follow up in LOPD1, 2, 3, 4, 5, 12, and 28.

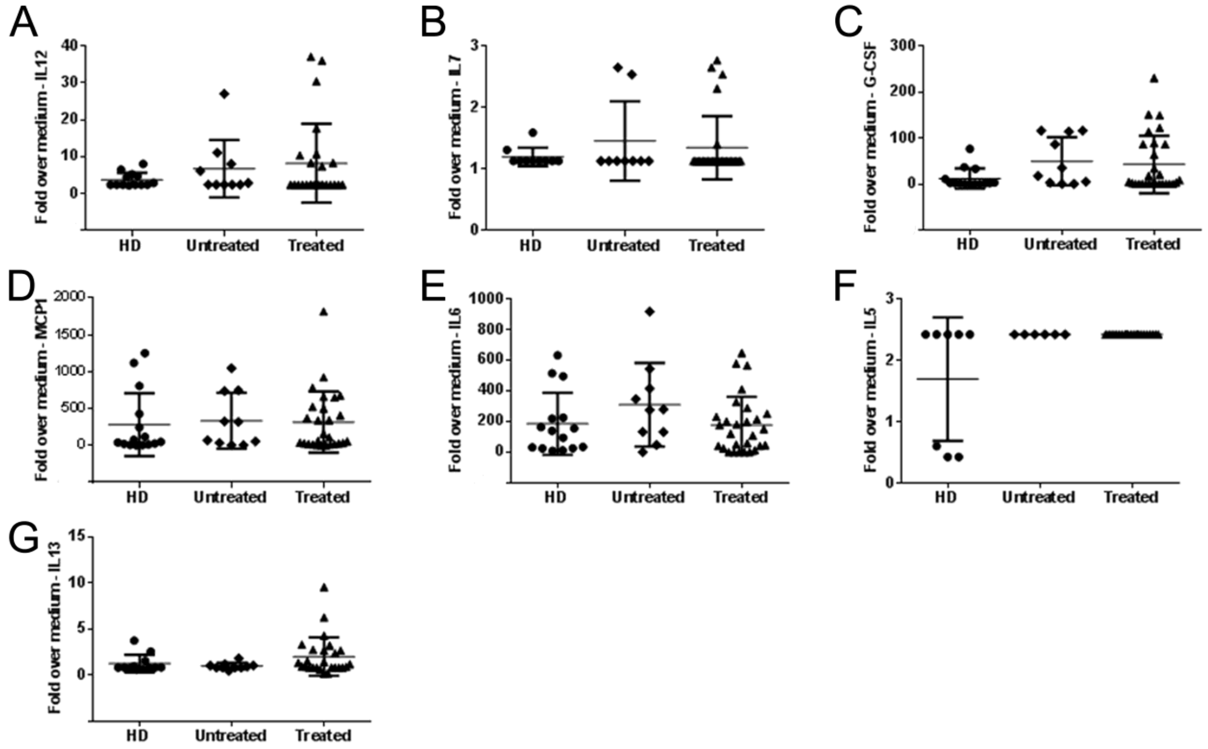

Supplementary Figure S2. Luminex assay for cytokine and chemokine production in supernatant of PBMCs restimulated with rhGAA. (A-G) Shown are levels of IL12, IL7, G-CSF, MCP1, IL6, IL5, and IL13. The Mann-Whitney test was used to compare data analysis across study groups.

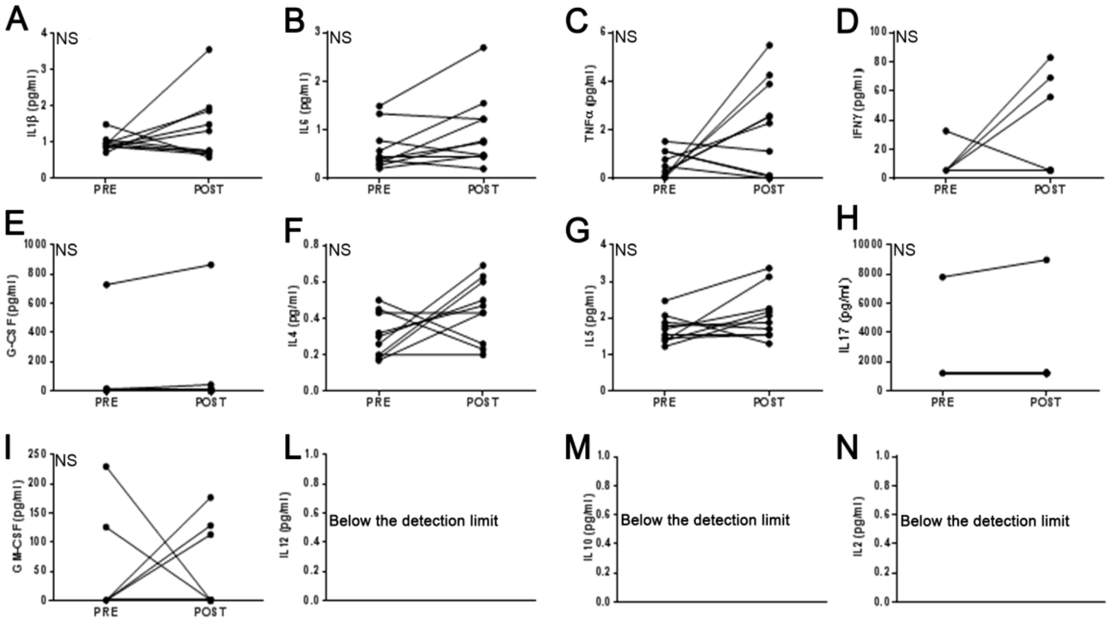

Supplementary Figure S3. Serum cytokine and chemokine profile of LOPD subjects receiving ERT. (A-N) Measurement of cytokines and chemokines in serum samples collected from each subject before (PRE) and at the end (POST) of rhGAA infusion. Paired t-test was used to compare data analysis PRE vs. POST infusion (NS, not significant).
